# Psychosocial Rehabilitation Interventions for Mental Health Disorders: A Systematic Review

**DOI:** 10.7759/cureus.116825

**Published:** 2026-09-24

**Authors:** Pooja Mohanty, Kirtikumar Rahul, Misbah Qureshi, Sharanya Sreekanthaswamy, Nikitha Sreekanthaswamy, Sijo Chacko

**Affiliations:** 1 Department of Psychotherapy and Counselling Services, Xavier School of Management (XLRI), Jamshedpur, IND; 2 Department of Community Medicine, Banas Medical College and Research Institute, Palanpur, IND; 3 Department of Forensic Medicine and Toxicology, Smt. Nathiba Hargovandas Lakhmichand Municipal Medical College (NHLMMC), Ahmedabad, IND; 4 Department of Medicine and Surgery, Kempegowda Institute of Medical Sciences (KIMS), Bengaluru, IND; 5 Department of Marketing, Dayananda Sagar Business Academy, Bengaluru, IND; 6 Department of Education, Oriental University, Indore, IND

**Keywords:** cognitive remediation, community mental health, mental health disorders, psychosocial rehabilitation, recovery-oriented care

## Abstract

Mental health disorders (MHD) contribute substantially to long-term disability and social exclusion, requiring interventions that extend beyond symptom reduction to recovery and functional outcomes. This systematic review evaluated psychosocial rehabilitation (PR) interventions for improving functioning, recovery, quality of life (QoL), and service-related outcomes among individuals with MHD. We conducted a structured search of PubMed/MEDLINE, Scopus, Web of Science, and PsycINFO for studies published between 2015 and 2025 using controlled vocabulary and free-text terms. Eligible studies evaluated PR-focused interventions in clinically diagnosed MHD and reported recovery, functioning, social participation, QoL, service engagement, self-management, or community integration outcomes. We excluded pharmacological-only studies, noninterventional studies, reviews, editorials, conference abstracts, and case reports. Eleven studies were included after screening 252 records. The evidence base comprised randomized and cluster-randomized trials, controlled intervention studies, secondary analyses, feasibility or exploratory studies, long-term follow-up evidence, and one clinical trial protocol, reflecting different levels of evidentiary strength. Although limited to 11 eligible studies, the review captured multiple PR models and outcome domains and was designed as a focused narrative synthesis rather than a definitive pooled comparison. Findings indicated potential benefits of cognitive remediation, CBT-integrated programs, Housing First, peer-led rehabilitation, and digital self-management for negative symptoms, social functioning, housing stability, recovery, and service utilization. Overall, PR appears valuable within recovery-oriented mental health systems, although heterogeneous designs and limited robust comparative evidence warrant cautious interpretation.

## Introduction and background

Mental health disorders (MHD) contribute substantially to long-term disability and sustained support needs across populations. In 2021, 14% of the global population, representing more than one billion individuals, were estimated to be living with a mental disorder, and mental disorders accounted for 5.2% of all disability-adjusted life years worldwide [1]. In schizophrenia, cognitive dysfunction is a major contributor to impaired social and occupational functioning and represents an important target for rehabilitation [2]. Psychosocial rehabilitation (PR) refers to a recovery-oriented approach that supports individuals with mental illness in developing the functional, social, vocational, and community-living skills required for meaningful participation and greater independence. For the purposes of this review, PR was operationalized as interventions primarily targeting recovery, everyday functioning, social participation, vocational functioning, self-management, service engagement, or community integration rather than symptom reduction alone.

PR has therefore developed as a structured approach aimed at improving participation, meaningful activity, vocational functioning, and community integration. The Clubhouse approach is a nonclinical, community-based rehabilitation model in which members and staff work collaboratively within a structured “work-ordered day,” emphasizing meaningful activity, social relationships, employment support, education, empowerment, and community participation [3]. Contemporary mental health systems increasingly emphasize recovery-oriented models that prioritize person-centered goals, empowerment, autonomy, and participation in meaningful social roles [4].

Community-based rehabilitation also requires coordinated support for individuals with severe and persistent mental illness. Intensive case management (CM) is a community-based model designed to provide coordinated, sustained, and individualized support for people with severe mental illness and complex service needs, with particular emphasis on maintaining engagement with services and reducing hospital use [5]. Intensive CM has been developed to improve continuity of care, service engagement, and management of complex support needs, with evidence suggesting reductions in hospital use in appropriately implemented programs [5]. Employment is another important component of recovery, and individual placement and support (IPS) is an evidence-based supported-employment model that helps individuals with severe mental illness obtain and sustain competitive employment through rapid job search, preference-based job matching, integration with mental health services, and ongoing support after employment [6].

Economic, labor-market, and regulatory conditions can influence the effectiveness of IPS, highlighting the importance of implementation context [6]. Cognitive remediation (CR) is a structured behavioral intervention designed to improve cognitive processes such as attention, memory, executive functioning, and problem-solving, with the broader aim of enhancing everyday functioning. It has also been extensively evaluated in schizophrenia, with meta-analytic evidence demonstrating improvements in cognitive performance and functional outcomes, particularly when CR is integrated with broader rehabilitation programs [7].

Methodological quality is an important consideration when interpreting rehabilitation evidence, and the Cochrane Risk of Bias 2 (RoB 2) framework provides a structured approach for evaluating potential bias in randomized trials [8]. Psychosocial and psychological interventions also contribute to relapse prevention in schizophrenia, with network meta-analytic evidence indicating that several intervention modalities can reduce relapse risk, although their comparative effectiveness varies [9]. Social skills training comprises structured behavioral and interpersonal exercises intended to strengthen communication, social interaction, and role functioning, often through practice-based methods such as role-play. Family psychoeducation provides structured information and support to relatives or caregivers to improve understanding of mental illness, coping, communication, and management of relapse-related burden. Housing First (HF) is a community-based rehabilitation model that provides immediate access to independent housing together with multidisciplinary mental health and recovery-oriented support, with housing stability and service utilization commonly assessed as outcomes. Peer-led rehabilitation involves recovery-oriented programs delivered or co-created by individuals with lived experience, with emphasis on empowerment, social participation, hope, self-esteem, and practical learning [10].

Illness Management and Recovery (IMR) is a structured recovery-oriented program that combines psychoeducation, goal setting, relapse-prevention strategies, and self-management techniques to help individuals understand and manage their illness and work toward personally meaningful recovery goals. Digital adaptations such as eHealth illness management and recovery (e-IMR) extend these principles through eHealth-supported delivery alongside face-to-face care. Meta-analytic evidence supports improvements in selected recovery and illness-management outcomes [10].

Community-based mental health services may improve clinical, social, and functional outcomes among individuals with severe mental illness, although effectiveness and cost-effectiveness vary according to service configuration and local context [11]. Broader reviews of community-based social interventions similarly support the value of coordinated rehabilitation approaches that promote participation, functioning, and sustained community living [12]. Adjunctive psychosocial modalities, including creative and expressive approaches such as music therapy, a structured therapeutic intervention that uses music-based activities and the therapeutic relationship to support psychological, emotional, cognitive, social, and functional outcomes, have also been evaluated across psychiatric and neurodevelopmental conditions, reflecting increasing interest in nonpharmacological rehabilitation strategies [13]. Unmet mental health needs also remain substantial across younger populations, reinforcing the importance of accessible rehabilitation-oriented services, although these populations were not a separate eligibility category in the present review [14].

These findings indicate that PR encompasses multiple complementary approaches, including cognitive rehabilitation, vocational support, CM, recovery-oriented services, psychosocial interventions, and community-based care. Differences in diagnostic populations, intervention components, outcome measures, and service settings can limit direct comparison across studies. Outcome assessment may focus on symptom reduction, functional status, social participation, quality of life (QoL), recovery indicators, employment, relapse, hospitalization, or other measures of service utilization. Previous reviews have largely evaluated individual components of PR separately, including supported employment, cognitive remediation, relapse-prevention interventions, Illness Management and Recovery, and community-based mental health services [6,7,9-12]. In particular, broad community-based reviews such as reference [12] have synthesized social and service interventions but have not provided an integrated comparison across the cognitive, vocational, family-focused, housing-based, peer-led, digital, and recovery-oriented PR models examined in the present review. Consequently, evidence remains fragmented across intervention types and outcome domains, with limited synthesis comparing how different PR models contribute to functional, recovery-oriented, quality-of-life, and service-related outcomes across mental health populations. This systematic review addresses this gap by integrating evidence across multiple PR approaches within a common framework to identify areas of consistent benefit, variation in effectiveness, and persistent evidence limitations.

Research question and PICO framework

The review question was as follows: Among individuals with clinically diagnosed MHD, including schizophrenia-spectrum disorders, bipolar disorder, depressive disorders, anxiety-related disorders, and other eligible conditions, how do PR interventions across cognitive, vocational, family-focused, case-management, housing-based, peer-led, digital, and recovery-oriented models, compared with usual care, alternative rehabilitation interventions, other reported comparators, or no comparator where permitted by study design, influence functional, recovery-related, quality-of-life, and service-related outcomes?

The review question was structured using a PICO framework [15]: Population (P): individuals with clinically diagnosed MHD, including schizophrenia-spectrum disorders, bipolar disorder, depressive disorders, anxiety-related disorders, and other eligible mental health conditions; Intervention (I): PR interventions addressing recovery, functioning, social participation, self-management, vocational functioning, service engagement, or community integration; Comparator (C): usual care, alternative psychosocial or rehabilitation interventions, other reported comparator conditions, or no comparator where permitted by the eligible study design; Outcomes (O): functional outcomes, recovery, QoL, social functioning, employment, adherence, relapse, hospitalization, emergency service use, and other relevant service-utilization outcomes. These elements are consistent with the eligibility criteria already specified in the manuscript.

Objectives of the review

The objective of this systematic review is to identify and synthesize evidence on PR interventions among individuals with clinically diagnosed MHD and to evaluate functional, recovery-related, QoL, and service-related outcomes across different rehabilitation models. Where comparators were available, outcomes were examined relative to usual care, alternative psychosocial or rehabilitation interventions, or other reported comparator conditions; noncomparative evidence was interpreted separately according to study design. The review also examines variation across intervention categories, populations, comparators, and implementation contexts to identify areas of consistent benefit, uncertainty, and persisting evidence gaps.

## Review

Methodology

Search Strategy

This systematic review was conducted to identify and synthesize evidence on PR interventions for individuals with MHD. We conducted a literature search across PubMed/MEDLINE, Scopus, Web of Science, and PsycINFO, with the final database search completed on August 20, 2026. We combined free-text keywords related to PR, recovery-oriented care, and severe mental illness with controlled vocabulary, including Medical Subject Headings, where applicable. The search concepts included “psychosocial rehabilitation”, “recovery-oriented care”, “mental health disorders”, “severe mental illness”, “cognitive remediation”, “social skills training”, “intensive case management”, “supported employment”, “Individual Placement and Support”, “family psychoeducation”, “Illness Management and Recovery”, “Housing First”, and “peer-led rehabilitation”. Relevant terms were combined using the Boolean operators AND and OR and adapted according to the indexing requirements of each database.

Searches were restricted to peer-reviewed studies involving human participants and published in English between January 2015 and December 2025. This period was selected to capture contemporary developments in recovery-oriented, community-based, vocational, cognitive, and digitally supported PR. The English-language restriction was predefined to permit consistent evaluation of eligible full-text publications. Study identification was based on the predefined electronic database search strategy, and potentially eligible records were assessed using the same prespecified eligibility criteria throughout the screening process.

Eligibility Criteria

Inclusion criteria: Eligible study designs included randomized controlled trials (RCTs) and cluster-randomized controlled trials, controlled intervention studies, observational or secondary analyses, and feasibility or exploratory intervention studies. Noncomparative studies were eligible when they evaluated a PR intervention and reported relevant rehabilitation or feasibility outcomes. Eligibility was based on the intervention addressing one or more prespecified PR domains, including recovery, functioning, social participation, self-management, vocational functioning, service engagement, or community integration. One protocol publication was retained separately to describe the intervention design and prespecified outcomes but was not considered completed effectiveness evidence. Accordingly, the evidence base comprised 10 completed studies and one protocol publication, and planned outcomes from the protocol were reported separately from observed intervention findings. No subsequent amendments to the eligibility criteria were made during the review process.

Exclusion criteria: Studies were excluded if they evaluated purely pharmacological interventions, were noninterventional, or did not report outcomes relevant to PR. Reviews, editorials, conference abstracts, case reports, and studies not published in English were excluded. Protocols without outcome data were excluded unless they were retained specifically to describe the design and planned outcomes of an intervention represented within the evidence base. Duplicate publications and studies for which full texts were unavailable were also excluded from the final selection.

Data Screening

Records identified through the database searches were screened in a stepwise manner according to the predefined eligibility criteria. After duplicate removal, two reviewers independently screened titles and abstracts, followed by independent full-text assessment of potentially eligible articles. Disagreements were resolved through discussion and consensus; when consensus could not be reached, a third reviewer adjudicated the decision. No automation tools were used during the screening process. The study-selection process was reported in accordance with the PRISMA 2020 framework and is summarized in the PRISMA flow diagram.

Data Extraction and Narrative Synthesis

Data from the included studies were independently extracted by two reviewers using a standardized data-extraction framework and were subsequently cross-checked for accuracy. Disagreements were resolved through discussion and consensus, with consultation of a third reviewer when required. No automation tools were used for data extraction. Extracted variables included author and year, country, study design, participant characteristics, diagnostic group, sample size, intervention and comparator, duration of follow-up, outcome measures, and key findings.

Outcomes of interest included functional outcomes, recovery outcomes, quality of life (QoL), social functioning, relapse, hospitalization, emergency service use, employment, adherence, and implementation-related outcomes where available. For each completed study, sample size, comparator, outcome instrument, follow-up duration, and quantitative findings were considered where reported. Within-group changes were distinguished from between-group comparisons, and both statistically significant and nonsignificant findings were retained. Protocol-only evidence was reported separately and was not interpreted as evidence of intervention effectiveness.

Due to heterogeneity in intervention components, participant populations, study settings, study designs, and outcome reporting, a quantitative meta-analysis was not conducted. Findings were synthesized narratively, grouped according to intervention category and outcome domain, and summarized in structured tables. Comparative randomized evidence was given greater interpretive weight than uncontrolled, qualitative, feasibility, or protocol-level evidence. Null and inconclusive findings were retained alongside beneficial findings to avoid overstatement of intervention effectiveness. Statements regarding improvement were linked to the specific outcome measure and analysis reported in the original study.

The review protocol was not prospectively registered with the International Prospective Register of Systematic Reviews or another systematic-review registry.

Quality Assessment and RoB Assessment

The methodological quality of the included studies was assessed to determine the strength and credibility of the evidence base. Two reviewers independently performed the methodological quality and risk-of-bias assessments. Disagreements were resolved through discussion and consensus, with consultation of a third reviewer when consensus could not be reached. No automation tools were used for these assessments.

RCTs were assessed using the Cochrane RoB 2 tool, version 22 August 2019 (Cochrane Bias Methods Group, Odense, Denmark), at the level of the principal rehabilitation outcome and the longest relevant follow-up time point reported for that outcome. Cluster-randomized trials were assessed using the cluster-randomized trial version of RoB 2, which additionally considers bias arising from identification or recruitment of participants within clusters. RoB 2 evaluates bias arising from the randomization process, deviations from intended interventions, missing outcome data, measurement of outcomes, and selection of the reported result [8].

Nonrandomized comparative intervention studies were assessed using the original 2016 Risk Of Bias In Non-randomized Studies of Interventions (ROBINS-I) tool, covering bias due to confounding, participant selection, classification of interventions, deviations from intended interventions, missing data, outcome measurement, and selection of the reported result. The original 2016 version is an established tool specifically designed for nonrandomized intervention studies.

Qualitative evidence, including the qualitative secondary analysis, was appraised using the Joanna Briggs Institute Critical Appraisal Checklist for Qualitative Research, considering methodological congruity, data collection and analysis, representation of participants, researcher influence, ethical considerations, and whether conclusions were supported by the analysis. For uncontrolled and feasibility intervention studies that did not correspond directly to RoB 2 or ROBINS-I comparative frameworks, methodological limitations were evaluated descriptively according to study design, including the presence or absence of a comparator, potential confounding, sample size, completeness of follow-up, reliance on self-reported outcomes, and the exploratory nature of the study. These studies were interpreted as preliminary feasibility or within-group evidence rather than comparative effectiveness evidence.

Individual judgments were supported by study-specific information concerning randomization and allocation procedures, masking, completeness of follow-up, outcome measurement, confounding, selective reporting, and other design-relevant limitations. Protocol-only evidence was not assigned an outcome-level risk-of-bias judgment because no completed outcome data were available. Risk-of-bias assessments directly informed the narrative synthesis: greater interpretive weight was given to studies with stronger methodological safeguards, whereas findings from studies with methodological concerns, noncomparative designs, or feasibility-stage evidence were interpreted cautiously and were not treated as equivalent to evidence from completed randomized trials. No studies were excluded solely on the basis of methodological quality.

Results

Study Selection

A total of 252 records were identified through the database searches. After removal of 41 duplicates, 211 unique records remained for screening. Following title and abstract screening, 164 records were excluded as not relevant to the review. The remaining 47 full-text articles were retrieved and assessed in detail for eligibility. Of these, 36 were excluded because they did not meet the inclusion criteria (n = 18), had insufficient outcome data (n = 13), or were published in a language other than English (n = 5). This selection process resulted in a final sample of 11 publications included in the systematic review, comprising 10 completed studies and one protocol publication. The PRISMA flow diagram summarizing the study-selection process is presented in Figure 1.

**Figure 1:**
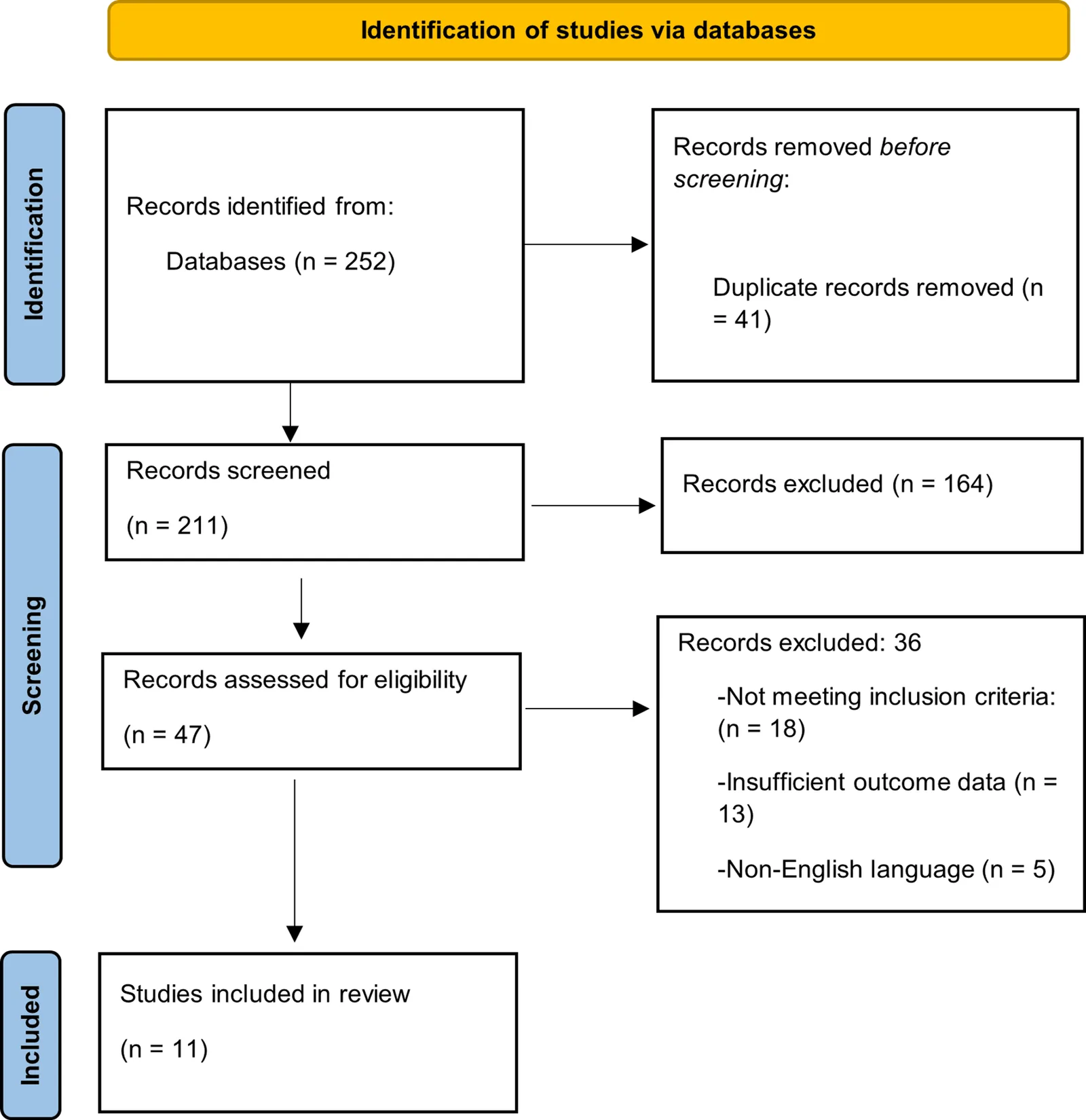
PRISMA flowchart

Characteristics of Included Studies

The included studies originated from multiple countries and service settings. The evidence base included randomized, cluster-randomized, feasibility, secondary-analysis, and protocol-based studies evaluating a range of PR approaches. The final evidence base comprised 11 publications, including 10 completed studies and one randomized clinical trial protocol without completed outcome data. The protocol was retained to describe the planned intervention and prespecified outcomes but was not considered evidence of clinical effectiveness. Cognitive remediation was represented by an RCT in first-episode schizophrenia [16]. The remaining completed studies evaluated interventions including Housing First (HF), brief family psychoeducation, integrated cognitive-behavioral and social-skills approaches, CM, illness management and recovery (IMR) programs, psychoeducation-based adherence interventions, integrated community care, peer-led rehabilitation, and vocational rehabilitation. The evidence therefore comprised studies of differing methodological strength and feasibility; qualitative/secondary and uncontrolled findings were interpreted separately from comparative randomized evidence. Table 1 describes the characteristics of the included publications, distinguishes completed effectiveness studies from protocol-only evidence, and summarizes the principal findings by study design and evidentiary context.

**Table 1:** Study characteristics and key findings of included psychosocial rehabilitation publications ACT: assertive community treatment; AIRIKI: “the power to love” in Japanese; a face-to-face, peer-led learning program about intimate and romantic relationships; CBSST-CCT: Cognitive Behavioral Social Skills Training-Compensatory Cognitive Training; CBT: cognitive behavioral therapy; CM: case management; CR: cognitive remediation; ED: emergency department; e-IMR: eHealth illness management and recovery; HBT: healthy behaviors training; HF: Housing First; IMR: illness management and recovery; IPS: individual placement and support; IPSE: individual placement and support supplemented with cognitive remediation and social-skills training; QALY: quality-adjusted life year; QoL: quality of life; VR-CBT: virtual reality-based cognitive behavioral therapy; CI: confidence interval; RCT: randomized controlled trial; RR: relative risk; SAU: service as usual; TAU: treatment as usual; ICER: incremental cost-effectiveness ratio

Study	Participants	Intervention details	Study design	Comparator	Main outcomes assessed	Key findings
Ventura et al. [16]	80 participants with first-episode schizophrenia; CR n = 31, HBT n = 49; mean age 21.5 years; 78% male	CR or HBT; 50 sessions over 6 months followed by booster sessions over the next 6 months	Randomized controlled trial	HBT	Negative symptoms; social functioning	CR significantly improved expressive and experiential negative symptoms and social functioning compared with HBT over 12 months
Christensen et al. [17]	720 adults with severe mental illness; IPS n = 243, IPSE n = 238, SAU n = 239; age 18-64 years; schizophrenia-spectrum, bipolar disorder, or recurrent depression	IPS alone or IPS supplemented with CR and social-skills training; 18-month follow-up	Randomized clinical trial-based economic evaluation	Service as usual	QALYs; employment/education; costs; cost-effectiveness	IPS and IPSE were significantly less costly than SAU by €9,543 and €7,288 per participant, respectively; participants also worked or studied more hours, although most ICER estimates were not statistically significant
Granholm et al. [18]	55 adults; CBSST-CCT n = 26, supportive contact n = 29; mean age 50.64 years; 60% male; schizophrenia n = 36, schizoaffective disorder n = 19	CBSST-CCT delivered as 25 twice-weekly 1-hour group sessions over 12.5 weeks	Pilot randomized controlled trial	Goal-focused supportive contact	Negative symptoms; cognition; functioning	CBSST-CCT significantly improved negative symptom severity and verbal learning compared with supportive contact; several functional outcomes did not show significant between-group improvement
Yasuma et al. [19]	83 family caregivers; intervention n = 43, TAU n = 40; mean age 69.4 vs. 67.4 years; female 65.1% vs. 75.0%	Brief family psychoeducation consisting of four 60-minute individual-family sessions completed over 1 month	Cluster randomized controlled trial	Treatment as usual	Caregiver burden (ZBI-22)	Caregiver burden decreased, but the between-group difference at 6 months was not significant (adjusted mean difference -2.12, 95% CI: -7.80 to 3.56; p = 0.45)
Schöttle et al. [20]	115 participants; schizophrenia-spectrum disorders n = 92 and bipolar I disorder n = 23	Integrated ACCESS care incorporating therapeutic ACT, delivered continuously with 48-month follow-up	Long-term nonrandomized observational follow-up	No concurrent control group	Psychopathology; functioning; QoL; service engagement; hospitalization	Significant within-group improvements were sustained over 4 years in psychopathology (d = 0.79), illness severity (d = 1.29), functioning (d = 0.77), and QoL (d = 0.47); findings lacked a concurrent comparator
Yamada et al. [21]	153 participants with schizophrenia; CM+TAU n = 78, TAU n = 75; median age 52 vs. 54 years; female 47.4% vs. 46.7%	CM comprising education and patient navigation for colorectal cancer screening; qualitative interviews evaluated acceptability and implementation	Qualitative secondary analysis of a mixed-method RCT	Treatment as usual in parent trial	Acceptability; appropriateness; feasibility; helpful components	Most responding patients considered the intervention acceptable, and providers considered it acceptable and appropriate; implementation for all patients simultaneously was considered difficult with available human resources
Levin et al. [22]	38 participants with bipolar disorder and hypertension; mean age 51.53 years; 53% women	Psychoeducation followed by 2 months of automated text messaging; educational/motivational and mood texts daily, with medication reminders added in month 2	Uncontrolled prospective intervention study	No concurrent comparison group	Medication adherence; systolic blood pressure; feasibility	Antihypertensive nonadherence decreased from 43% to 21% and systolic blood pressure from 144.8 to 136.0 mmHg at 12 weeks (both p<0.001); causal effectiveness cannot be established because there was no control group
Beentjes et al. [23]	60 participants with severe mental illness; e-IMR+IMR n = 41, IMR n = 19; mean age 46.9 vs. 40.7 years; female 73.2% vs. 31.6%	Weekly 2-hour face-to-face IMR sessions using 11 modules, with the intervention group additionally receiving web-based e-IMR; outcomes assessed to 12 months	Exploratory cluster randomized controlled trial	Face-to-face IMR alone	Self-management; recovery; symptoms; QoL; general health	Initial improvements in selected outcomes were affected by confounding and interaction effects; only 56.1% logged into e-IMR and overall effectiveness could not be established
Kageyama et al. [24]	45 participants with mental disorders; mean age approximately 40 years; 57.8% male	AIRIKI, a face-to-face peer-led group program on intimate and romantic relationships using structured learning, role-play, and communication activities	One-group pretest-posttest mixed-method feasibility study	No control group	Recovery; hope; self-esteem; communication; feasibility	Significant within-group changes were observed in recovery, hope, self-esteem, and selected communication measures; findings remain preliminary because of the uncontrolled feasibility design
Jeppesen et al. [25]	Planned n = 256 participants with schizophrenia-spectrum disorders	10 sessions of VR-CBT plus treatment as usual vs. 10 sessions of standard CBT plus treatment as usual	Randomized assessor-blinded clinical trial protocol	Standard CBT + treatment as usual	Planned paranoid ideation; social anxiety; safety behaviors; social functioning; QoL	Protocol publication only; no completed outcome data were reported and no effectiveness conclusions were drawn
Tinland et al. [26]	703 homeless adults with severe mental illness; HF n = 353, TAU n = 350; mean age 38.1 vs. 39.4 years; male 80.2% vs. 84.9%; schizophrenia or bipolar disorder	Immediate Housing First placement with recovery-oriented multidisciplinary support; 24-month follow-up	Treatment as usual	Hospital admissions; ED visits; inpatient days; housing stability; QoL	HF significantly reduced inpatient days (51.8 vs. 83.6 days; RR: 0.62, 95% CI: 0.42-0.80; p < 0.001) and improved housing stability, with no significant advantage in hospital admissions or ED visits.	-

Classification of PR Interventions

Cognitive and cognitive-behavioral rehabilitation: AIRIKI, meaning “the power to love” in Japanese, is a face-to-face, peer-led learning program focused on intimate and romantic relationships that uses structured group learning, role-play, and communication-based activities to strengthen interpersonal skills [24]. Cognitive and cognitive behavioral therapy (CBT)-oriented rehabilitation interventions included CR, integrated CBT-based psychosocial skills rehabilitation, and a Virtual Reality-based Cognitive Behavioral Therapy (VR-CBT) clinical trial protocol [25]. The VR-CBT publication described the planned trial design and prespecified outcomes, including paranoid ideation, social anxiety, safety behaviors, social functioning, and QoL, rather than reporting completed clinical outcomes.

Social and skills-based interventions: Social and skills-based rehabilitation was represented primarily by peer-led learning interventions. AIRIKI was delivered in small, face-to-face group sessions incorporating structured modules and role-play scenarios to strengthen communication and relationship-related social skills [24].

CM and assertive community approaches: Community-based intensive support models included HF with assertive community treatment (ACT) support and integrated care models incorporating therapeutic ACT. In the included HF study [26], immediate access to independent housing was combined with multidisciplinary mental health support. The ACT component was based on the established ACT model [27]. The intervention was evaluated using inpatient days, emergency department (ED) visits, hospitalization, recovery, QoL, housing stability, and costs [26].

Recovery-oriented and illness management programs: Recovery-oriented and illness management programs included IMR-related interventions and peer co-created rehabilitation programs. AIRIKI assessed recovery using the Recovery Assessment Scale [28], together with measures of hope and self-esteem as core recovery-related outcomes [24].

Community and housing-based rehabilitation: Housing-based PR was represented by the HF RCT conducted in France [26]. Housing stability was assessed over 24 months using measures including stably housed days and the Residential Time-Line Follow-Back method [26]. The classification of PR interventions by subsection category is presented in Table 2.

**Table 2:** Classification of PR interventions across included studies ACT: assertive community treatment; AIRIKI: “the power to love” in Japanese, a face-to-face peer-led learning program about intimate and romantic relationships; CBT: cognitive behavioral therapy; CR: cognitive remediation; e-IMR: eHealth illness management and recovery; IMR: illness management and recovery; IPS: individual placement and support; QoL: quality of life; PR: psychosocial rehabilitation; VR-CBT: virtual reality-based cognitive behavioral therapy; HF: Housing First; CM: case management; QALY: quality-adjusted life year

Intervention classification	Intervention model identified	Description	References
Cognitive and cognitive-behavioral rehabilitation	CR; integrated CBT-based PR; VR-CBT	Structured cognitive and behavioral rehabilitation approaches targeting cognition, negative symptoms, social functioning, and related psychosocial outcomes; VR-CBT is represented by protocol-level evidence only	[16,18,25]
Social and skills-based interventions	Peer-led skills learning (AIRIKI)	Face-to-face peer-led group learning using role-play and communication-based activities to strengthen interpersonal and relationship-related skills	[24]
CM and assertive community approaches	HF + ACT; integrated care including therapeutic ACT; CM support	Community-based models providing coordinated, sustained support to improve service engagement, participation, continuity of care, and community functioning	[20,21,26]
Recovery-oriented and illness management programs	e-IMR; peer co-created recovery learning; psychoeducation + adherence support	Recovery- and self-management-oriented interventions delivered through digital augmentation, peer-led learning, psychoeducation, and adherence support	[22-24]
Community and housing-based rehabilitation	HF recovery-oriented housing support	Immediate access to independent housing combined with multidisciplinary recovery-oriented support and community integration services	[26]
Economic and vocational rehabilitation	IPS; IPS supplemented with CR and social-skills training	Vocational rehabilitation approaches aimed at competitive employment, with economic evaluation of employment, QALY, cost, and cost-effectiveness outcomes	[17]
Family-focused PR	Brief family psychoeducation	Structured psychoeducation delivered to family caregivers through psychiatric visiting-nurse services to support coping, communication, and management of caregiver burden	[19]

Outcomes of PR

Functional outcomes: Functional outcomes were reported across several completed studies and included social functioning, communication-related abilities, employment-related functioning, and housing stability. In the CR trial, greater improvement in selected negative-symptom domains and social functioning was reported relative to HBT [16]. Integrated CBT social-skills training combined with compensatory cognitive training also improved selected negative-symptom and cognitive outcomes, although several functional outcomes did not differ significantly between groups [18]. AIRIKI reported within-group improvements in communication-related abilities, including communicating about oneself and listening to others; these findings were derived from a one-group feasibility study and should be interpreted as preliminary noncomparative evidence [24].

Clinical and symptom outcomes: Clinical and service-related outcomes included negative symptoms, hospitalization, ED visits, inpatient days, and related clinical measures. The CR and integrated CBT-based studies reported improvements in selected negative-symptom outcomes [16,18]. In the HF RCT, no significant between-group differences were observed in hospital admissions or ED visits, whereas inpatient days were significantly lower in the HF group than in the treatment-as-usual group [26]. Findings from the e-IMR study were mixed and did not establish clear effectiveness because observed effects were influenced by confounding, interaction effects, and low platform use [23].

Recovery and QoL: Recovery and QoL outcomes were assessed across several interventions. HF measured recovery using the Recovery Assessment Scale [28] and assessed QoL using disorder-specific and generic instruments, including the Schizophrenia Quality of Life Questionnaire-18 [29] and 36-Item Short Form Health Survey [30]. HF was associated with improvement in selected QoL domains, including psychological well-being and autonomy [26,29]. AIRIKI demonstrated within-group changes in recovery, hope, and self-esteem, with significant improvements in selected measures at one-month follow-up; these findings represent feasibility-stage evidence rather than comparative effectiveness [24]. The uncontrolled psychoeducation and text-messaging intervention also reported within-group adherence and clinical changes, but the absence of a concurrent comparator limits causal interpretation [22]. Table 3 presents the synthesis of functional, recovery-related, and service-related outcomes across the included interventions.

**Table 3:** Synthesis of functional and recovery outcomes across included studies CR: cognitive remediation; IPS: individual placement and support; CBT: cognitive behavioral therapy; CCT: compensatory cognitive training; ACT: assertive community treatment; e-IMR: eHealth illness management and recovery; AIRIKI: “the power to love” in Japanese, a face-to-face peer-led learning program about intimate and romantic relationships; VR-CBT: virtual reality-based cognitive behavioral therapy; QoL: quality of life; QALY: quality-adjusted life year; CM: case management; ED: emergency department; HBT: healthy behaviors training; HF: Housing First; RCT: randomized controlled trial

Intervention category	Functional outcomes	Recovery outcomes	Service-related outcomes	Evidence interpretation	References
CR	Greater improvement in selected negative-symptom domains and social functioning compared with HBT	Functional benefit was associated with cognitive improvement	Not the primary focus	Comparative RCT evidence	[16]
IPS/IPSE	Participants worked or studied more hours than those receiving service as usual	QALY outcomes favored the supplemented intervention in some analyses	Lower costs and favorable cost-effectiveness findings were reported	RCT-based economic evidence; some estimates were not statistically significant	[17]
Integrated CBT social skills + CCT	Significant improvement in negative symptoms and selected cognitive outcomes; several functional outcomes were not significantly different between groups	Psychosocial functioning outcomes were assessed, with mixed between-group findings	Not the primary focus	Pilot comparative RCT evidence	[18]
Brief family psychoeducation	No direct patient functional outcome was the primary endpoint	Caregiver burden decreased over time, but between-group differences were not statistically significant	Not the primary focus	Cluster RCT evidence with a null between-group primary finding	[19]
Integrated care with ACT (ACCESS II)	Long-term improvements in functioning were observed during follow-up	Improvements in psychopathology, illness severity, and QoL were sustained over four years	Low disengagement and hospitalization rates were reported	Long-term observational follow-up without a concurrent comparator; causal inference is limited	[20]
CM-based screening participation support	Engagement with preventive health services was explored	Acceptability, appropriateness, and implementation experiences were reported	Participation-related implementation outcomes were described	Qualitative secondary-analysis evidence; not comparative effectiveness evidence	[21]
Psychoeducation + text messaging	Within-group improvement in medication adherence was observed	Self-management-related changes were reported	Clinical adherence and blood-pressure outcomes improved during follow-up	Uncontrolled prospective evidence; causal effectiveness cannot be established	[22]
e-IMR	Selected self-management and recovery-related outcomes improved in initial analyses	Recovery-related changes were reported, but findings were affected by confounding and interaction effects	Platform use was low, and clear service-level benefit was not established	Exploratory cluster RCT evidence; overall effectiveness remained inconclusive	[23]
Peer-led learning (AIRIKI)	Improvements were observed in selected communication-related abilities	Within-group improvements in recovery, hope, and self-esteem were reported, with significant changes in selected measures	Not the primary focus	One-group feasibility evidence; preliminary and noncomparative	[24]
VR-CBT vs. standard CBT	Social functioning was specified as a planned secondary outcome	Paranoid ideation was the planned primary outcome; no completed recovery outcomes were reported	Not assessed as completed effectiveness evidence	Protocol-level evidence only; planned outcomes, not measured effects	[25]
HF + recovery-oriented support	Greater housing stability and autonomy-related benefits were reported	Improvements were observed in selected QoL domains	No significant differences in hospital admissions or ED visits; fewer inpatient days were reported	Comparative RCT evidence with both beneficial and null findings	[26]

Protocol-level planned outcomes: The VR-CBT publication [25] was a randomized clinical trial protocol and was therefore reported separately from completed effectiveness evidence. The protocol specified paranoid ideation as the planned primary outcome and included social anxiety, safety behaviors, psychosocial functioning assessed using the Personal and Social Performance scale, and QoL among the planned secondary outcomes. No completed clinical outcome data were available, and these outcomes were not interpreted as measured intervention effects.

Service utilization and economic outcomes: Service utilization outcomes included ED visits, hospital admissions, inpatient days, and economic costs. In the HF study, the intervention was associated with lower overall costs, with a mean between-group difference of €217 per patient over 24 months in favor of HF, alongside reported savings in healthcare and residential service costs [26].

Synthesis of findings

Due to heterogeneity in intervention categories, outcome measures, and study designs, findings from the 11 included studies were synthesized narratively. Outcomes were organized into functional, clinical and service-related, recovery-related, and economic domains. Because the evidence base included controlled trials, secondary analyses, feasibility studies, and protocol-level evidence, structured tabulation was used to facilitate comparison across intervention models and outcome domains (Figure 2). Individual studies could contribute to more than one outcome domain when multiple categories of outcomes were reported; therefore, the frequencies presented in Figure 2 are not mutually exclusive and should not be summed to represent the total number of included studies.

**Figure 2:**
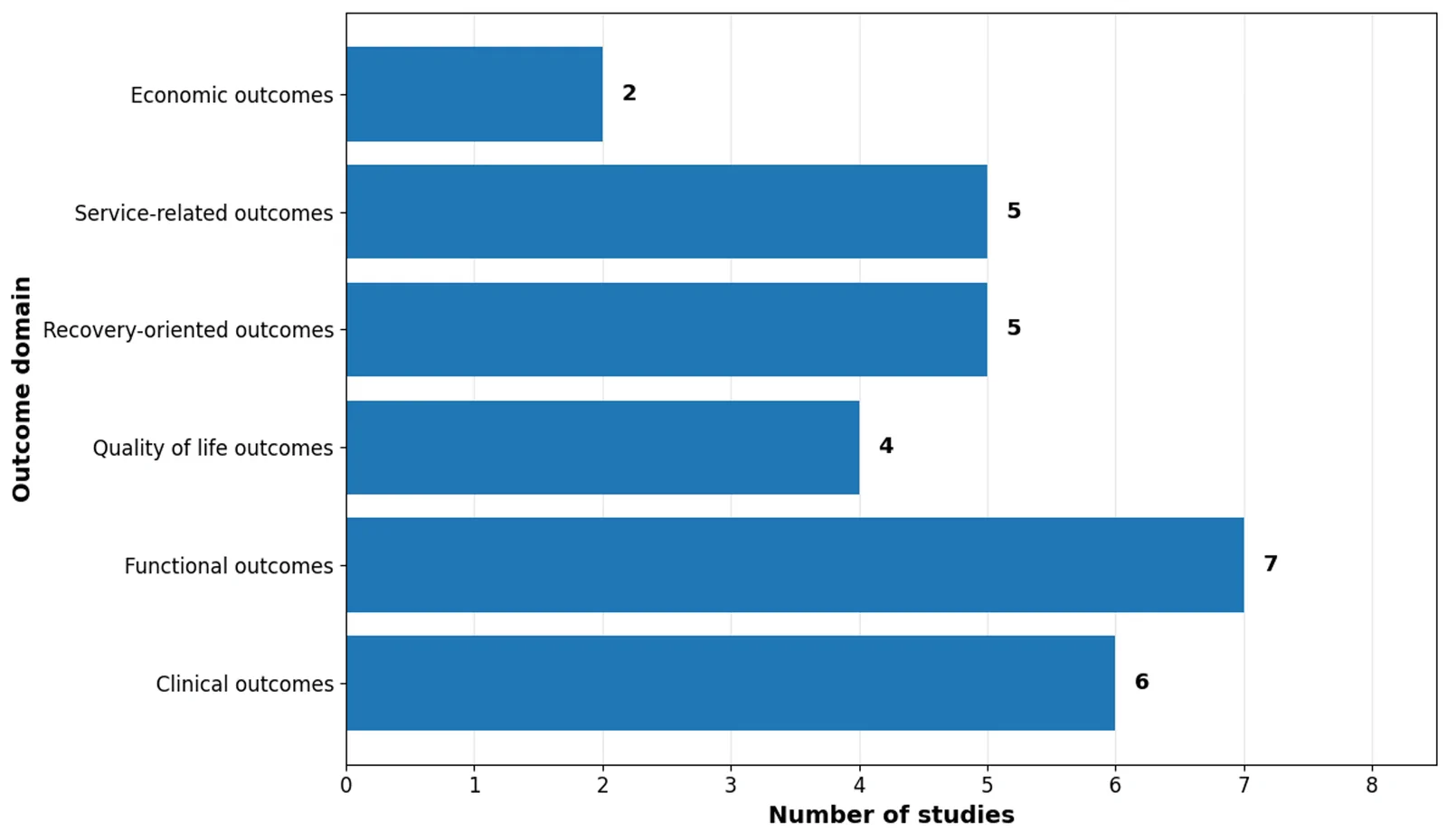
Outcome domains reported across completed included studies Economic outcomes were reported in two completed studies [17,26], service-related outcomes in five [17,20-22,26], recovery-oriented outcomes in five [20,22-24,26], quality-of-life outcomes in four [17,20,23,26], functional outcomes in seven [16-18,20,23,24,26], and clinical outcomes in six [16,18,20,22,23,26]. Individual studies could contribute to more than one domain. The VR-CBT protocol [25] was excluded from these frequencies because it reported planned outcomes only and no completed outcome data Image credit: This is an original image created by the author Pooja Mohanty using Google Colab (Google LLC, Mountain View, CA) VR-CBT: virtual reality-based cognitive behavioral therapy

Quality Assessment Findings

The included evidence comprised studies with varying levels of methodological rigor. Randomized designs were represented by the HF trial [26] and the brief family psychoeducation cluster RCT [19]. The AIRIKI intervention used a one-group pretest-posttest feasibility design and therefore provided feasibility-stage evidence rather than comparative effectiveness evidence [24]. The VR-CBT publication was a planned randomized clinical trial protocol and did not report completed intervention outcomes [25].

RoB Assessment

RoB was assessed according to study design and the methodological safeguards reported in each study. For randomized studies, judgments considered the RoB 2 domains of the randomization process, deviations from intended interventions, missing outcome data, measurement of outcomes, and selection of the reported result [8]. Cluster-randomized trials were assessed with consideration of cluster-specific risks, including recruitment and allocation at the cluster level. Nonrandomized studies were evaluated using design-relevant considerations including participant selection, confounding, intervention classification, missing data, outcome measurement, and selective reporting. Uncontrolled and feasibility studies were interpreted separately from comparative effectiveness studies, with particular caution regarding confounding and the absence of a concurrent comparator. The included studies showed varying levels of methodological concern across these domains, particularly regarding masking, allocation procedures, follow-up completeness, and outcome assessment.

In the HF trial, participants and interviewers were unmasked, raising concerns regarding outcome measurement for self-reported outcomes [26]. The brief family psychoeducation cluster trial used cluster randomization and intention-to-treat analysis, reported attrition below 20%, and achieved full intervention adherence, supporting lower concern for missing outcome data and deviations from intended interventions [19]. Levin et al. [22] was an uncontrolled prospective intervention study and was therefore not assessed as a controlled comparative study; its findings were interpreted cautiously because the absence of a concurrent comparator limits causal inference. The qualitative secondary analysis and feasibility-stage evidence were also interpreted according to their respective study designs and were not assigned the same evidentiary weight as completed randomized trials. Protocol-only evidence was not assigned an outcome-level risk-of-bias judgment because completed outcome data were unavailable. The complete risk-of-bias assessments are presented in Table 4.

**Table 4:** Risk of bias assessment of included studies JBI: Joanna Briggs Institute; RCT: randomized controlled trial; RoB 2: risk of bias 2; ROBINS-I: Risk Of Bias In Non-randomized Studies of Interventions

Study	Study design	Appraisal tool	Main methodological considerations	Overall assessment
Ventura et al. [16]	RCT	RoB 2	Randomization adequately described; low concern for missing data; some concern related to blinding and outcome measurement	Some concerns
Christensen et al. [17]	RCT-based economic evaluation	RoB 2	Based on randomized allocation; low concern for missing outcome data; some concern related to outcome measurement and deviations from intended interventions	Some concerns
Granholm et al. [18]	Pilot RCT	RoB 2	Randomized design with a limited sample size; some concern related to blinding and measurement of subjective outcomes	Some concerns
Yasuma et al. [19]	Cluster RCT	Cluster-RCT RoB 2	Cluster randomization; low attrition; complete intervention adherence; some concern related to outcome measurement	Some concerns
Schöttle et al. [20]	Long-term nonrandomized follow-up study	ROBINS-I	No concurrent comparator at long-term follow-up; potential confounding and selection bias; some concern related to outcome measurement	Moderate methodological concern
Yamada et al. [21]	Qualitative secondary analysis of a mixed-methods RCT	JBI critical appraisal checklist for qualitative research	Secondary qualitative analysis; interpretation based on participant and provider accounts and qualitative data collection	Some methodological concerns
Levin et al. [22]	Uncontrolled prospective intervention study	Design-appropriate noncomparative appraisal	No concurrent comparator; potential confounding; causal intervention effects cannot be established	Some methodological concerns
Beentjes et al. [23]	Exploratory cluster RCT	Cluster-RCT RoB 2	Exploratory design; possible confounding and interaction effects; low use of the eHealth platform; some concern related to missing data and outcome measurement	Some concerns
Kageyama et al. [24]	One-group pretest-posttest feasibility study	Design-appropriate feasibility appraisal	No control group; small feasibility sample; reliance on within-group and self-reported outcomes	Some methodological concerns
Jeppesen et al. [25]	Randomized clinical trial protocol	Not applicable	No completed outcome data available for outcome-level risk-of-bias assessment	Not assessed
Tinland et al. [26]	RCT	RoB 2	Randomized design; participants and interviewers were unmasked; some concern related to the measurement of subjective outcomes	Some concerns

Discussion

The present review synthesized findings from 11 studies evaluating different PR interventions across severe mental health conditions. Cognitive remediation (CR) and integrated CBT-based approaches showed improvements in negative symptoms and social functioning [16,18]. Evidence from the e-IMR study [23] was inconclusive; although some recovery-related changes were observed, the apparent effects were sensitive to confounding and interaction effects, platform use was low, and the investigators concluded that effectiveness could not be established. In contrast, the peer-led AIRIKI feasibility study [24] reported within-group improvements in recovery, hope, and self-esteem, including significant changes in selected measures at one-month follow-up; these findings should be interpreted as preliminary feasibility evidence rather than comparative effectiveness evidence. HF with recovery-oriented support was associated with improved housing stability, fewer inpatient days, and benefits in selected quality-of-life (QoL) domains [26]. Technology-supported interventions, including digital IMR and text-based adherence models, showed potential to complement conventional rehabilitation delivery, although evidence for the effectiveness of e-IMR remained inconclusive [22,23]. Family psychoeducation and integrated community-care models addressed caregiver, service-engagement, and recovery-related outcomes [19,20]. Overall, the included studies emphasized the multidimensional role of PR across functional, recovery-oriented, and service-related domains.

The broader pattern of findings is consistent with evidence indicating that comprehensive early-intervention models may be cost-effective while supporting functional outcomes in psychosis [31]. Long-term integrated care incorporating therapeutic ACT has also demonstrated sustained clinical and service-related benefits in severe schizophrenia-spectrum and bipolar disorders [20]. Evidence from mobile-supported adherence interventions [32] and self-management interventions for persistent depressive disorder [33] was used as contextual literature rather than as part of the 11-study evidence base. These studies illustrate how rehabilitation-related principles may extend across different service settings and diagnostic groups. These findings support integrating PR within broader recovery-oriented mental health systems [4].

CR findings from the current review are consistent with meta-analytic evidence demonstrating improvements in cognitive performance and associated functional outcomes in schizophrenia [7]. Cognitive dysfunction is closely associated with functional disability in schizophrenia, reinforcing the importance of targeted rehabilitation strategies [2]. Social-skills and CBT-integrated approaches are also consistent with evidence showing improvements in interpersonal and social functioning in psychosis [18,34]. Relapse prevention remains an important rehabilitation objective, and psychosocial and psychological interventions have been associated with reduced relapse risk in schizophrenia [9]. These findings support cognitive and behavioral rehabilitation as important components of comprehensive PR.

Housing-based rehabilitation findings in the current review highlight the importance of stable accommodation and community integration in recovery [26]. Community-based mental health services have also demonstrated benefits in social and functional outcomes, although their effectiveness varies according to service configuration and contextual factors [11]. Economic evaluations indicate that labor-market conditions, regulatory environments, and service infrastructure may influence vocational rehabilitation outcomes [6]. These contextual determinants are consistent with recovery-oriented models that conceptualize rehabilitation as a coordinated system-level process rather than an isolated intervention [4]. The findings therefore suggest potential value in delivering PR through organized community-based systems of care, although the comparative effectiveness of individual models remains uncertain.

Family psychoeducation findings in the included evidence support the broader role of psychosocial interventions in maintaining stability and reducing relapse-related burden in schizophrenia [9]. IMR programs and their digital adaptations are consistent with meta-analytic evidence demonstrating improvements in self-management and selected recovery outcomes [10]. Peer-led recovery initiatives, including co-created learning models, reflect rehabilitation approaches that emphasize empowerment, participation, and lived-experience expertise [3,24]. These approaches support person-centered rehabilitation aligned with service-user priorities, including relationships, self-esteem, social participation, and community belonging.

Across the included evidence, PR was operationalized through multiple modalities spanning cognition, social skills, housing, family systems, CM, vocational support, and digital delivery. The multidimensional nature of these approaches reflects the complex functional impairment associated with severe mental illness [2]. Broader evidence concerning unmet mental health needs provides contextual support for accessible rehabilitation-oriented services across diverse populations and settings [14]. Within the included evidence, rehabilitation interventions were represented primarily across schizophrenia-spectrum and bipolar disorders [20]. Evidence from depressive disorders [33], mobile-supported adherence interventions [32], and comparative social-skills approaches [34] was used only as contextual supporting literature and was not counted among the 11 included studies. These references were cited to place the included findings within the broader rehabilitation literature rather than to characterize the included evidence. Taken together, the included studies and supporting literature indicate that PR may support selected recovery-related outcomes, including real-world functioning, participation, and sustained community living [4,12].

Limitations and Future Recommendations

The included evidence was heterogeneous in intervention content, outcome measurement, study design, and follow-up duration, limiting direct comparison between PR models. Several studies used feasibility, exploratory, secondary-analysis, or protocol designs, while others focused predominantly on service-related rather than standardized recovery outcomes. Variation in participant populations, care settings, intervention delivery, comparator conditions, and outcome-assessment procedures further limited consistency across studies. Some interventions lacked robust comparator conditions or blinded outcome assessment, increasing the potential for bias, particularly for subjective outcomes. The inclusion of protocol-level evidence also limited conclusions regarding clinical effectiveness because completed outcome data were unavailable for that intervention. The predominance of evidence from schizophrenia-spectrum and other severe mental illness populations also limits generalizability across the broader range of MHD included in the eligibility framework.

Future research should prioritize adequately powered randomized trials using harmonized functional, recovery, and QoL measures, together with longer follow-up to assess sustainability of benefit. Comparative-effectiveness studies across core rehabilitation models, accompanied by implementation research, would help identify which intervention components contribute most strongly to outcomes in routine clinical practice. Greater representation of diverse diagnostic groups, low-resource settings, and culturally adapted service models is also needed to improve generalizability and support scalable rehabilitation policy and practice.

## Conclusions

This systematic review synthesized evidence from 11 publications comprising 10 completed studies and one protocol publication evaluating PR interventions across MHD, with a primary focus on severe mental illness. The findings suggest that selected PR approaches may improve functional, recovery-related, quality-of-life, and service outcomes, although the certainty of these findings is limited by the small evidence base, substantial heterogeneity in study designs and interventions, and methodological concerns. Cognitive remediation and integrated CBT-based approaches showed benefits in selected negative-symptom and social-functioning outcomes. HF was associated with improved housing stability, fewer inpatient days, and benefits in selected quality-of-life domains, but did not demonstrate significant advantages in hospital admissions, ED visits, or recovery outcomes. Peer-led interventions showed preliminary within-group improvements in hope, self-esteem, and recovery-related measures, but these findings were derived from feasibility and noncomparative evidence. Digital and mobile-supported interventions produced mixed findings; e-IMR effectiveness remained inconclusive, and uncontrolled improvements in adherence-related outcomes cannot establish causality. Overall, the available evidence does not establish the comparative effectiveness of different PR models. Findings should therefore be interpreted cautiously, particularly given the inclusion of randomized, secondary-analysis, feasibility, uncontrolled, and protocol-level evidence. Further adequately powered comparative studies using standardized outcome measures, longer follow-up, and consistent reporting of effect estimates are needed to determine the relative effectiveness and sustainability of individual PR approaches.
